# 2DMatPedia, an open computational database of two-dimensional materials from top-down and bottom-up approaches

**DOI:** 10.1038/s41597-019-0097-3

**Published:** 2019-06-12

**Authors:** Jun Zhou, Lei Shen, Miguel Dias Costa, Kristin A. Persson, Shyue Ping Ong, Patrick Huck, Yunhao Lu, Xiaoyang Ma, Yiming Chen, Hanmei Tang, Yuan Ping Feng

**Affiliations:** 1 Department of Physics, National University of Singapore, Singapore, 117551 Singapore; 2 Department of Mechanical Engineering, National University of Singapore, Singapore, 117575 Singapore; 3 Centre for Advanced Two-dimensional Materials, National University of Singapore, Singapore, 117546 Singapore; 4 Department of Materials Science and Engineering, University of California Berkeley, California, 94720 USA; 5 Lawrence Berkeley National Laboratory, Berkeley, California 94720 USA; 6 Department of NanoEngineering, University of California San Diego, 9500 Gilman Drive, La Jolla, California 92093 USA; 7 State Key Laboratory of Silicon Materials, School of Materials Science and Engineering, Zhejiang University, Hangzhou, 310027 China

**Keywords:** Theory and computation, Electronic properties and materials

## Abstract

Two-dimensional (2D) materials have been a hot research topic in the last decade, due to novel fundamental physics in the reduced dimension and appealing applications. Systematic discovery of functional 2D materials has been the focus of many studies. Here, we present a large dataset of 2D materials, with more than 6,000 monolayer structures, obtained from both top-down and bottom-up discovery procedures. First, we screened all bulk materials in the database of Materials Project for layered structures by a topology-based algorithm and theoretically exfoliated them into monolayers. Then, we generated new 2D materials by chemical substitution of elements in known 2D materials by others from the same group in the periodic table. The structural, electronic and energetic properties of these 2D materials are consistently calculated, to provide a starting point for further material screening, data mining, data analysis and artificial intelligence applications. We present the details of computational methodology, data record and technical validation of our publicly available data (http://www.2dmatpedia.org/).

## Abstract (summary)

**Table:** 

Design Type(s)	modeling and simulation objective • material transformation objective
Measurement Type(s)	material entity
Technology Type(s)	digital curation
Factor Type(s)	Approach • element • material property
Sample Characteristic(s)

Machine-accessible metadata file describing the reported data (ISA-Tab format)

## Background & Summary

Atomically thin two-dimensional (2D) materials have attracted tremendous research interest for both novel fundamental physics and extremely appealing applications. For example, new emerging physics such as half-integer quantum Hall effect^1^, Klein tunnelling^2^, valley Hall effect^3^ and topological superconductivity^4,5^ have been reported in 2D materials. 2D structures are naturally beneficial for performance of various types of devices, such as large surface-to-volume ratio for high sensing sensitivity and catalysis efficiency^6,7^, reduced size for immunity against short channel effects^8^ and flexibility for wearable devices^9,10^, to name only a few. Furthermore, the Van-der-Waals stacking of homo/hetero 2D materials provides another degree of freedom to tune the desired properties of the system^11,12^. However, only dozens of 2D materials have been experimentally synthesized. New 2D materials with novel properties are needed to meet the demand of ever growing technological applications.

The traditional material discovery is mainly based on trial-and-error experiments which are time consuming and resource intensive. To accelerate development and deployment of novel advanced materials, the US White House launched the “Materials Genome Initiative” in 2011 (https://www.mgi.gov/). This approach integrates high throughput computation, data analytics together with experimental research and represents a new paradigm for materials discovery. The data-driven material discovery can significantly reduce the cost from many long iterations of trial-and-error experiments by providing the most promising candidates from high-throughput computations. This approach is also more flexible as different screenings can be conducted to target materials with specific properties for different applications. In this spirit, large repositories with millions of computed bulk material entries have been developed such as the Materials Project (MP)^13^, the Open Quantum Materials Database (OQMD)^14,15^, the Automatic Flow for Materials Discovery (AFLOWLIB)^16^, and the Novel Materials Discovery (NOMAD, http://nomad-repository.eu; https://nomad-coe.eu) Laboratory, thanks to the development of computing power and significant advancements of the accuracy of first-principles calculations^17^.

Databases specific for 2D materials have also emerged rapidly from small repositories with tens of entries within some specific structure prototypes in earlier works^18–22^ to more recent systematic data mining of the Materials Project, Inorganic Crystal Structure Database (ICSD, http://www2.fiz-karlsruhe.de/icsd_home.html) and the Crystallographic Open Database (COD)^23^, yielding thousands of monolayers^24–27^. Nevertheless, most of the current databases for 2D materials have been obtained mainly using the top-down approach, that is, by theoretically exfoliating monolayers from layered bulk materials. And some criteria on the stability and exfoliation energy were applied to predict potentially exfoliatable stable monolayers^24,26^. This method allows a systematic screening of 3D materials for layered structures. However, the top-down approach is limited in at least three cases. (i) Some 2D materials, such as silicene^28^, do not have corresponding layered bulk forms. (ii) A 2D material, such as MoS_2_^29^, can have a few stable polymorphs but only one of them has a corresponding bulk form. (iii) Existing 3D material databases are not comprehensive enough and some known materials are not captured in these databases. For example, the bulk form of the seminal 2D ferromagnetic CrI_3_ was unfortunately absent in Materials Project^13^. On the other side, chemical substitution has been shown to be an efficient way to predict new 2D materials^21,30^. Furthermore, 2D materials with relatively high exfoliation energies can be grown by other methods than mechanical exfoliation, and metastable 2D materials can be stabilized by means such as been supported on a substrate^28,31^.

Here, we present a comprehensive 2D Materials Encyclopedia (2DMatPedia) database, with monolayers obtained from both the top-down and the bottom-up approaches. We screen all the possible layered bulk materials from the Materials Project by a topology-based algorithm, and theoretically exfoliate them into monolayers. Then, we systematically generate new 2D materials by elemental substitution of the unary and binary compounds obtained from the top-down approach. This combined top-down and bottom-up approach has generated more than 6,000 2D structures. Their structural, electronic and energetic properties have been obtained by high-throughput calculations. This large and consistent 2D material dataset could serve as a good starting point for further materials screening, data mining and training of machine learning (ML) models. The whole database is publicly available at http://www.2dmatpedia.org/.

## Methods

**Fig. 1:**
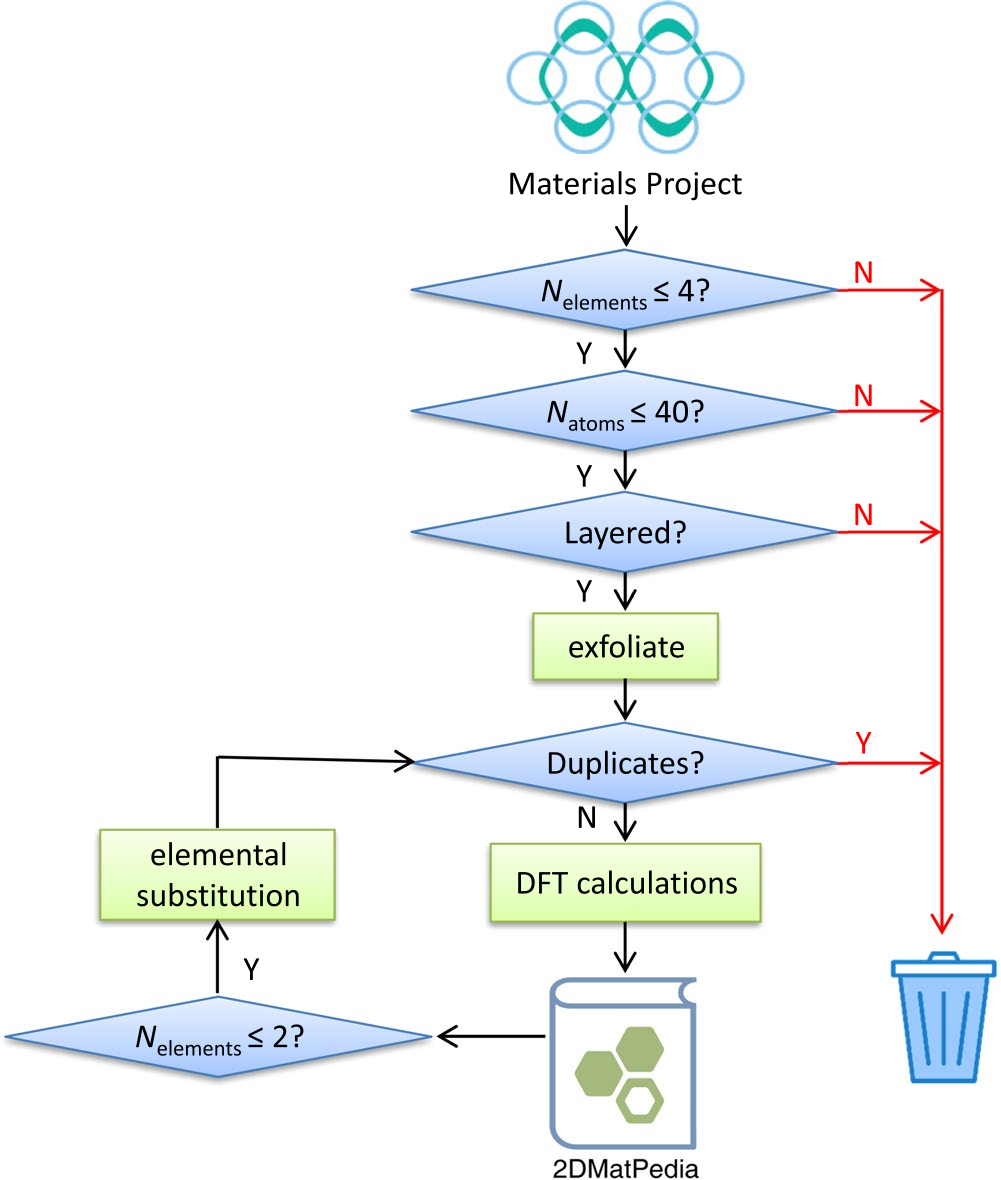
The workflow of producing data of 2D materials.

The methodology applied in this work mainly includes two parts: discovery processes to generate 2D structures and high-throughput density functional calculations of properties of the 2D materials. The overall workflow is shown in Fig. 1 and the details of each step are explained below.

### Discovery processes

We use both a top-down approach, in which materials from the inorganic bulk crystals in the Materials Project are screened for layered structures which are then theoretically exfoliated to 2D monolayers, and a bottom-up approach, in which elemental substitution is systematically applied to the unary and binary 2D materials obtained from the top-down approach. It is noted that the “discovery process” here only indicates how a 2D material is generated in this work, which is not necessarily related to its experimental synthesis method. For example, 2D CrI_3_ as mentioned above is generated by the bottom-up approach here, but was exfoliated experimentally^32^.

### Top-down approach

A geometry-based algorithm^24,26^ was used to identify layered structures among the bulk materials in Materials Project, by the following steps:

1. The conventional unit cell was used for all the compounds.
2. Check whether any pair of atoms in the unit cell are bonded, using the sum of the covalent radii^33^ of the two elements with a small tolerance as a threshold. If the distance between the two atoms is smaller than the threshold, they are considered bonded. Otherwise, they are not bonded.
3. Atoms that are bonded together form a cluster. The number of clusters in the unit cell is counted.
4. From the unit cell, a 3 × 3 × 3 supercell is generated and the number of clusters in the supercell is counted again. If the number of clusters in the supercell is three times of that in the unit cell, the structure was tagged as layered.
5. A set of tolerances (0.0, 0.05, 0.1, 0.15, 0.2, 0.25, 0.3, 0.35, and 0.4) were used and only the structures identified as layered by at least two tolerances are kept.
6. 2D materials were theoretically exfoliated by extracting one cluster in the standard conventional unit cell of the layered structures screened in the above steps. A vacuum of at least 20 Å along the direction perpendicular to the 2D layer (*c* axis) was imposed to minimize the interactions of image slabs by periodic condition.

### Bottom-up approach

1. All the elements of the periodic table (from H to Bi) are categorized in different groups according to their column number. Radioactive elements, lanthanide and actinide with *f* electrons are excluded except La which is included in group 3 elements.
2. Systematically replace each element in a known 2D material by one other element in the same group. For instance, 24 new materials can be generated from BN by all the possible combination of the first element from group [B, Al, Ga, In, Tl] and second one from [N, P, As, Sb, Bi].

### Workflow

As shown in Fig. 1, we started from the >80000 inorganic compounds in Materials Project database. In the initial stage, we focus on elemental, binary, ternary and quaternary compounds and also ignore structures with more than 40 atoms in the primitive unit cell. The top-down approach discussed above was applied to screen the database for layered structures and generate 2D materials. Structure matching tools from pymatgen^34^ were used to remove duplicates in the exfoliated 2D materials. High-throughput calculations, adopting the same standards set by the Materials project, were carried out to optimize the structures, and perform total energy, density of states (DOS) and band structure calculations for these 2D materials. The calculated properties are stored in 2DMatPedia. The unary and binary 2D materials obtained from the top-down approach were then used as initial structures for elemental substitution. Structure matching was applied again to these 2D materials obtained through this bottom-up approach to ensure that only unique structures are included for further high-throughput density functional theory (DFT) calculations.

### High-throughput calculations

The standard workflow^35,36^ developed by Materials Project is used to perform high-throughput calculations for all the layered bulk and 2D materials generated in the above processes. The DFT calculations are performed using the Vienna Ab initio Simulation Package (VASP) with the frozen-core all-electron projector-augmented wave (PAW) method for the electron-ion interaction^37,38^. All calculations are performed with spin polarization and high initial magnetic moments for magnetic ions. Calibrated Hubbard U values are applied for the transition metals ions in transition metal oxides and fluorides^39–41^. The cutoff energy for the plane wave expansion of electron wavefunction is set to 520 eV. The interlayer dispersion interaction in layered materials is included via the dispersion-corrected vdW-optB88 exchange-correlation functional^42–45^. This functional has been shown to reproduce reasonably well the results from the more accurate but computationally demanding random-phase approximation (RPA) calculations^24,46^. It is noted that dipole corrections are not applied for the compounds in this dataset. Users interested in properties that are more sensitive to dipole corrections are advised to re-optimize the polar structures to get more accurate results.

### Structure optimization

For structure optimization of 2D materials, both cell shape and volume are adjusted (ISIF = 3) while the *c* axis is kept fixed. The energy difference for ionic convergence is set to 1.0 × 10^−4^ eV. The dispersion-corrected vdW-optB88 exchange-correlation functional is applied for structure relaxation.

### DOS and band structure calculations

A static run with a uniform, Γ-centered *k*-point grid for the relaxed structure is performed first to generate the charge density. Non-self-consistent runs based on the charge density with an energy range from *E*_F_ (Fermi level) − 10 eV to *E*_F_ + 10 eV with 2000 intervals are used for DOS simulation. The band structure computation uses line-mode k-point grid along the high symmetry points of the 2D Brillouin zone^24,47^. Van der Waals correction is not applied in these calculations.

### Exfoliation energy and decomposition energy

The exfoliation energy, the average energy per atom required to remove a layer from a layered bulk material, is calculated from by *E*_exf_ = *E*_2D_ − E_bulk_, where *E*_2D_ and *E*_bulk_ are the total energy per atom of the 2D and its layered bulk counterpart, respectively. To obtain the total energy, similar structure optimization and static calculations are performed for both the 2D and bulk materials. The total energy of the static run is used to calculate the exfoliation energy. The dispersion-corrected vdW-optB88 exchange-correlation functional is applied to all the runs in the exfoliation energy calculations.

However, not every 2D material has a unique bulk counterpart. To ensure a meaningful comparison of exfoliation energies of various 2D materials, the following assumptions are made. If a 2D material has only one corresponding layered bulk in the Materials Project, this bulk material is tagged as its layered bulk counterpart. If there are multiple layered bulk structures in MP corresponding to a given 2D material, only the one with the lowest energy is tagged as its layered bulk counterpart. For 2D materials obtained through the bottom-up approach, there is often no existing corresponding layered bulk material. In such a case, we also construct a “layered bulk” counterpart by stacking the monolayers following the same stacking sequence in the initial structure (i.e., BN in the example given earlier). It is noted that such a “layered bulk” material is unlikely to be the most stable bulk counterpart for the 2D material obtained by the bottom-up approach, especially for a few special cases like Silicene, which do not have layered bulk counterparts. For these compounds, the exfoliation energies can be significantly underestimated and are provided only for reference. The energetic stability of such a 2D material is better estimated by the decomposition energy as discussed below.

The decomposition energy is defined as the energy required to separate a compound into its components. Here, we applied a modified version of the energy above hull, and define the decomposition energy as the energy required (per atom) to decompose a given material into the set of most stable materials at this chemical composition^48^. This definition of decomposition energy excludes the corresponding layered bulk of the given 2D material from its competing phases. The energy difference between a 2D material and its layered bulk is given by the exfoliation energy.

The competing entries are queried from Materials Project, which are calculated without the Van der Waals correction. In order to make direct comparison, we also calculate the total energies of 2D materials without the Van der Waals correction. This energy and the energies of all entries belonging to the same chemical system in Materials Project are used to generate a phase diagram. The decomposition energy is obtained from the difference between the energy of the 2D material and those of its competing phases.

## Data Records

The web interface of 2DMatPedia, implemented using Flamyngo (https://materialsvirtuallab.github.io/flamyngo/), can be found at http://www.2dmatpedia.org/. A JSON file is available on the web interface (http://www.2dmatpedia.org/download) and also in a Figshare repository^49^. Table 1 shows the key variables of this materials collection, including name, data type and a short description. The ‘material_id’ is the identifier of the each unique 2D material in the dataset. The ‘relative_id’ provides easy link to the material from which this 2D material is obtained. The ‘discovery_process’ shows how the 2D material is generated (“top-down” or “bottom-up”). The ‘structure’ is the relaxed structure in a dictionary format. It can be easily transferred to different format by pymatgen. Other information that can be deduced from a structure, such as chemical formula (‘formula’), number of elements in the structure (‘nelements’), list of elements (‘elements’), ‘ spacegroup’, ‘ point_group’, are also listed for easy query. The documented electronic, energetic and magnetic properties are ‘bandgap’, ‘is_gap_direct’, ‘is_metal’, ‘energy_per_atom’, ‘energy_vdw_per_atom’, ‘exfoliation_energy_per_atom’, ‘decomposition_energy_per_atom’ and ‘total_magnetization”.

**Table 1:** JSON keys for metadata and their descriptions.

Key	Datatype	Description
material_id	string	IDs for entries in the 2Dmatpedia
relative_id	string	IDs for where a 2D material is obtained from
discovery_process	string	How a 2D materials is generated
structure	dictionary	Relaxed crystal structure represented in dictionary
formula	string	Chemical formula
nelements	string	Number of elements in this material
elements	list	List of elements in this material
spacegroup	string	Space group number defined by The International Union of Crystallography
point_group	string	Point group in Hermann-Mauguin notation
bandgap	float	Energy band gap of this material
is_gap_direct	Boolean	Is the material a direct gap
is_metal	Boolean	Is the material metallic
energy_per_atom	float	Energy per atom in eV without vdW correction
energy_vdw_per_atom	float	Energy per atom in eV with vdW correction
exfoliation_energy_per_atom	float	Exfoliation energy of the 2D material in eV/atom
decomposition_energy_per_atom	float	Decomposition energy of the 2D material in eV/atom
total_magnetization	float	Total magnetic moment in *μ*_B_

**Fig. 2:**
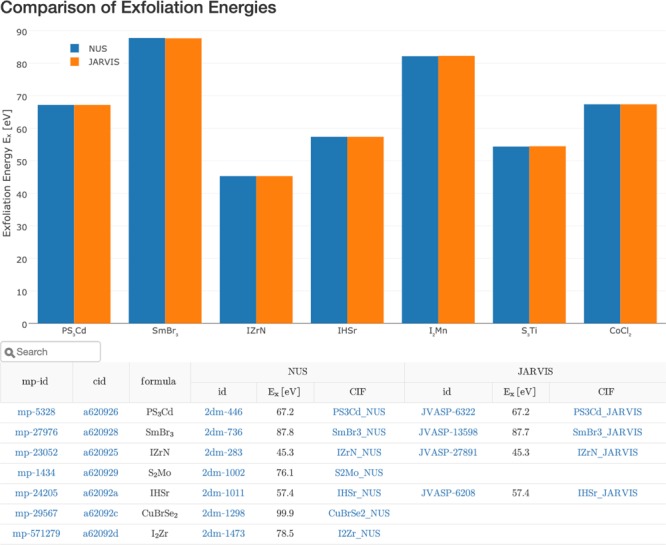
Comparison of exfoliation energies from 2DMatPedia with NIST’s JARVIS. A table in the interactive MPContribs landing page links each MP material to the according entries in the 2DMatPedia and JARVIS-DFT websites.

The exfoliation energies and calculated structures have also been contributed to Materials Project. Figure 2 shows the dataset’s landing page (https://materialsproject.org/mpcontribs/jarvis_dft/) on the MPContribs Portal^50^. It interactively compares exfoliation energies from 2DMatPedia to the DFT section of NIST’s Joint Automated Repository for Various Integrated Simulations (JARVIS)^25,51^. An overview table directly links materials details pages on MP to the according details pages on 2DMatPedia and JARVIS-DFT. Crystallographic Information Files (CIFs) for structures contributed by the two projects are also provided for download. The MP materials details pages expose the contributed data via an embedded preview to a community of >70 k materials scientists. This allows 2DMatPedia’s data to be discovered by MP users using its programmatic and web search interfaces when they query the portal for properties of specific materials. A streamlined MPContribs pipeline has also been set up for 2DMatPedia collaborators to continuously update the data presented on Materials Project.

## Technical Validation

We have performed the following validations for our results in 2DMatPedia. First, we benchmark the structures and energies against data in an existing 2D material database, JARVIS, which applied well-converged energy cut-off and k-mesh density. Then we analyse the calculated decomposition energies for 59 experimentally grown 2D materials.

### Convergence

We apply the standard workflow designed by the Materials project for two reasons. (1) The parameters for energy cutoff, k-point mesh, and threshold of energy convergence are extensively tested by the Materials project (https://materialsproject.org/docs/calculations). (2) It makes our results compatible with the entries in the Materials project, and we could make direct comparisons to get useful information such as decomposition energy. To evaluate the performance of these standard workflow in predicting structures and energies of 2D materials, we compare the calculated lattice parameters and exfoliation energies of the subset of materials (~400 materials out of >6,000) that are also present in the JARVIS database^52^.

**Fig. 3:**
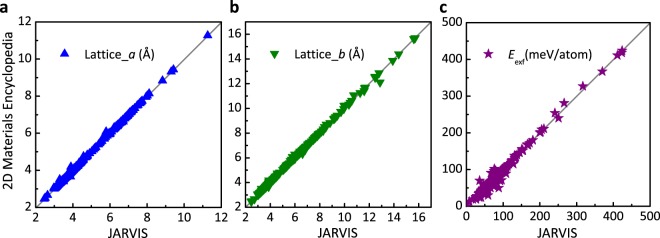
Structural and energetic comparison of the results from JARVIS and in this work. (**a**–**c**) Are the comparisons for lattice constants of *a*, *b*, and exfoliation energy respectively.

As shown in Fig. 3, the calculations in this work reproduce very well the results in JARVIS and the differences in lattice constants and exfoliation energies between 2DMatPedia and JARVIS are very small. The mean error and standard deviation are 0.009 Å and 0.034 for lattice parameter *a*, 0.010 Å and 0.062 for lattice parameter *b*, −1.13 meV/atom and 5.64 for exfoliation energy, respectively. Among the 383 data collected, 93% of them have an exfoliation energy difference smaller than 10 meV/atom.

### Decomposition energy

**Fig. 4:**
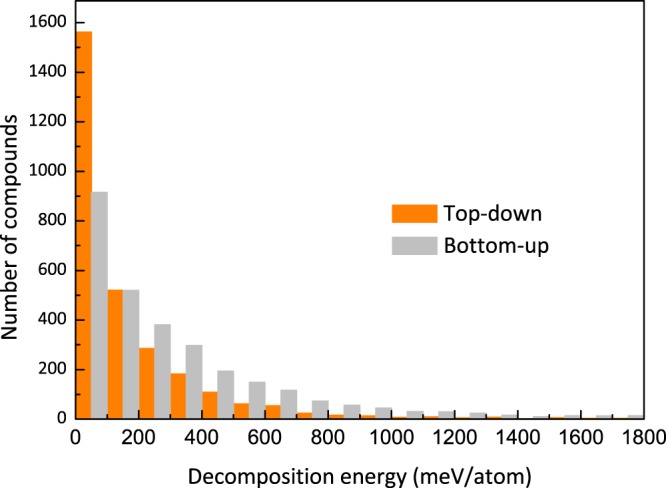
Histogram of the decomposition energy for both the top-down and bottom-up 2D materials in this work.

Figure 4 shows the histogram of decomposition energy for 2D materials obtained from both top-down and bottom-up approaches in 2DMatPedia. Of the 2,884 2D materials obtained via the top-down, more than 1,500 could be energetically stable, with a low decomposition energy of less than 100 meV/atom, while for those obtained through the bottom-up design, around 900 out of 2,927 are considered stable. The overall distribution shows that the number of compounds obtained from the top-down approach drops very fast with the decomposition energy, and almost vanishes at 1600 meV/atom, while those obtained from bottom-up approach decreases slowly and has significant number of compounds even at 1800 eV/atom. This can be understood by that top-down approach starts from the existing bulk materials while the bottom-up compounds are generated by artificial elemental substitution. Nevertheless, we keep these less stable 2D structures in our database, as such structures may have good functionalities and they could be stabilized by other means such as by a substrate. It is noted that the bottom-up approach starts from the abundant combinations of element groups from the top-down 2D materials, and thus a same-column elements substitution as applied here allows us to cover most of possible compositions. The large number of relative stable 2D compounds obtained from the bottom-up approach shows its efficiency in massive production of new stable 2D materials, which plays an important, complementary role to the top-down method in generating 2D structures to build a more comprehensive 2D materials dataset.

**Fig. 5:**
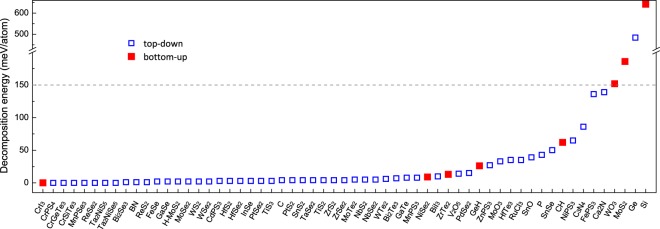
Decomposition energy calculated in this work for 59 experimentally grown 2D materials. The blue open squares are 2D materials generated in a top-down approach while the red solid squares are the ones from the bottom-up approach in this work.

Figure 5 shows the calculated decomposition energy for the 59 experimentally grown 2D materials^28,31,32,53–99^. It is noted that 53 of them have decomposition energies of less than 100 meV/atom and 39 compounds within 10 meV/atom. The 4 compounds with a relatively high value (>150 meV/atom) – 2D WO_3_, T′-MoS_2_, Ge (germanene), Si (silicene) – are known to be metastable in free standing form but have been synthesized on metal substrates or by metal atom intercalation^28,64,71,99^. Ca_2_N has a high exfoliation energy (260 meV/atom in this work which is in good agreement with result of previous work^58^) but has been exfoliated into 2D nanosheets by liquid exfoliation and suspension in organic solvents.

In Fig. 5, compounds discovered by the top-down approach are indicated by blue open squares and those obtained by the bottom-up approach are shown in red solid squares. It is obvious that the top-down approach alone failed to capture some of the experimentally grown compounds. And the bottom-up generated 2D materials such as Silicene, 1 T′-MoS_2_ and CrI_3_ demonstrate its complementary role to the top-down approach in the discovery of 2D materials.

Overall, our calculations have yielded reasonable values of decomposition energy for 2D materials, and this quantity can be used as a guide for experimental synthesis of the 2D materials.

### Band gap

**Fig. 6:**
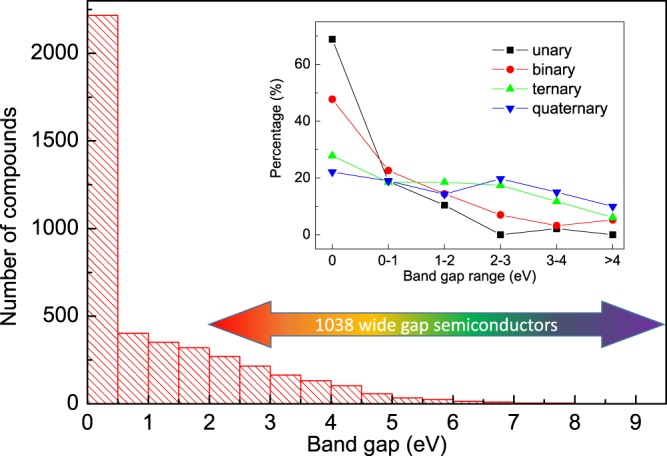
Statistical analysis of the band gaps for the relatively stable (decomposition energy <0.6 eV/atom) 2D materials in 2DMatPedia. The main panel shows a histogram of number of compounds in the different band gap ranges. The inset presents detailed distribution of the number of compounds within different band gap ranges for the unary, binary, ternary and quaternary compounds, respectively. The number in the legend denotes the group number of elements.

Recently, wide-band-gap (>2 eV) 2D materials have sparked some interest for potential applications in optoelectronic devices working under the blue or UV light^100–102^. To demonstrate the capability of the 2DMatPedia, we performed a statistic analysis on the band gaps (*E*_g_) of the relatively stable (decomposition energy <0.6 eV/atom) 2D materials in 2DMatPedia. Even though the DFT (GGA-PBE) calculations are known to underestimate the band gaps, such an analysis can not only provide a general idea on the ratio of compounds that are good candidates for the wide-band-gap 2D materials but also provide information on the hidden trends, which are valuable for designing new wide-band-gap 2D materials. As shown in Fig. 6, there are around 1000 monolayers with band gap larger than 2 eV among the 4328 relatively stable 2D materials. Considering the relatively fewer studies on wide-band-gap 2D materials, this dataset with a large number of candidates presents a good base for further investigations. In addition, the distribution of the number of compounds within different band gap ranges shown in the inset of Fig. 6 demonstrates some interesting trends. Overall, the number of compounds within the band gap range decreases with increase in band-gap. Most (>45%) of the unary and binary compounds are metallic and their numbers within the band gap range drop much faster than ternary and quaternary compounds. Only around 2% of the unary compounds while around 45% of the quaternary ones are wider band-gap (*E*_*g*_ > 2 eV) 2D materials. This suggests that ternary and quaternary 2D compounds are more likely to have wide band gaps than unary and binary ones.

## Usage Notes

The dataset in this work presents a large collection of >6000 2D materials with consistently calculated structural, energetic and electronic properties. Currently, the database includes unary and binary bottom-up 2D compounds generated by systematic elemental substitution. Such a method could be directly applied to ternary and quaternary compounds, but due to the large design space, it would not be the most efficient method. We are developing machine learning algorithms to perform a preliminary screening of the elemental substituted ternary and quaternary 2D materials to exclude unstable compounds and then perform high-throughput calculations for the remaining materials with reasonable stability. A similar process will also be applied to a cross-column element substitution. And the database is also growing with new 2D materials by compounds from literature and 2D alloys. The user will be able to scan the entire database to screen for 2D materials with new functionalities, data mining, data analysis, and artificial intelligence applications.

## ISA-Tab metadata file

Download metadata file

## Data Availability

The code used in this work relies heavily on the open-source tools developed by Materials Project, in particular, Python Materials Genomics (http://pymatgen.org/) and Atomate (https://atomate.org). The versions used in this work are pymatgen/4.7.3 and atomate/0.5.1 for all the calculations.
